# Mechanical Stimulation of Equine Bone Marrow Mesenchymal Stromal Cell-Derived Cartilage-Like In Vitro Model Triggers Osteoarthritis Features

**DOI:** 10.1021/acsbiomaterials.5c00500

**Published:** 2025-06-13

**Authors:** Romain Contentin, Cassie Jehl, Kevin Commenchail, Florence Legendre, Philippe Galéra, Frédéric Cassé, Magali Demoor

**Affiliations:** 27003 Université Caen Normandie, Normandie Univ, BIOTARGEN UR7450, Normandie Equine Valée, GIS CENTAURE, F-14000 Caen, France

**Keywords:** osteoarthritis, equine cartilage, mechanical compression, *in vitro* model, mesenchymal stromal cells, chondrocytes

## Abstract

Osteoarthritis (OA) affects millions of people globally, causing irreversible cartilage damage, chronic inflammation, and progressive joint dysfunction. Similarly, horses can develop OA spontaneously or due to their athletic careers, influenced by mechanical and biochemical factors. Current treatments primarily focus on symptom relief without promoting cartilage regeneration. In line with the 3Rs principles (refine, reduce, replace), the development of *in vitro* OA models is essential for advancing new therapeutic approaches against OA. In response to this need, the present study aimed to develop an *in vitro* model of mechanically induced OA. Bone marrow-derived mesenchymal stromal cells (BM-MSCs) were cultured in a biomaterial scaffold and differentiated for 21 days using a chondrogenic medium to produce cartilage-like *in vitro* models. The cartilage-like *in vitro* models underwent mechanical stimulation (compression) for 3 and 7 days at pressures sufficient to induce injurious stress. BM-MSC-derived chondrocytes express the transient receptor potential vanilloid-type 4 (TRPV4) channel and are responsive to mechanical stimulation. Mechanical stimulation was found to reduce cell proliferation without inducing cell death. The overall protein levels of type II collagen drastically declined after both 3 and 7 days of mechanical stimulation. Additionally, glycosaminoglycan (GAG) content within the cartilage-like *in vitro* models decreased, whereas GAG release into the supernatant increased following mechanical stimulation. Ultimately, compression led to the upregulation of catabolic factors and inflammatory mediators. In conclusion, this model successfully replicates several key features of OA, making it a valuable tool for investigating the disease’s mechanisms and testing new therapeutic strategies.

## Graphical abstract

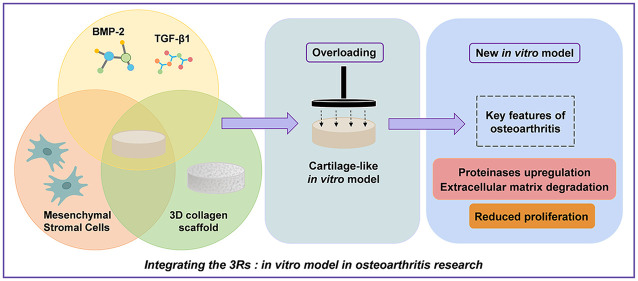

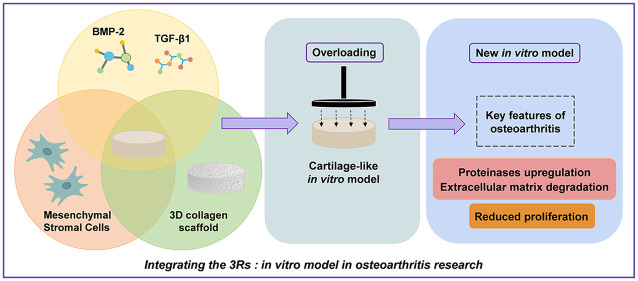

## 1 Introduction

Osteoarthritis (OA) is a prevalent condition affecting millions worldwide, particularly impacting both humans and horses. This debilitating disorder is characterized by irreversible cartilage degradation, chronic inflammation, and progressive decline in joint function, driven by both mechanical and biochemical factors.^1^ Beyond the economic impact, OA is a leading cause of chronic pain, long-term disability, and deterioration both in humans and in animal welfare.^1–4^ While current treatments focus on alleviating symptoms, they fail to regenerate damaged cartilage, emphasizing the need for models that accurately replicate OA. Developing such models is crucial for advancing therapeutic strategies, as they enable a deeper understanding of disease progression and open avenues for discovering treatments that address the underlying causes of cartilage degradation.^5^

Joint injury is a complex process, typically involving high amplitudes and multifaceted loading modes that trigger biological cell signaling pathways involved in articular cartilage degradation.^6,7^ Given the limited innate capacity of cartilage to repair itself, traumatic injuries that lead to the loss of chondrocyte viability and extracellular matrix components often result in permanent damage. The upregulation and increased activity of degrading enzymes such as A disintegrin and metalloproteinase with thrombospondin motifs (ADAMTS), matrix metalloproteinases (MMP), and high-temperature requirement protein A1 (HTRA1) lead to the degradation of the cartilage extracellular matrix (ECM).^8^ This increased catabolism releases various damage-associated molecular patterns (DAMPs), promoting the expression of inflammatory mediators and exacerbating catabolism.^9^

Mechanical loading plays a critical role in cartilage homeostasis regulated by chondrocytes and has been shown to influence cellular behavior.^10,11^ However, injurious loading can have detrimental effects on cartilage. Studies conducted *in vitro* have shown that excessive compression is associated with increased cell death, including necrosis and apoptosis,^12–14^ the release of proteoglycans, and the initiation of ECM degeneration.^12,15^ Although the progression of OA *in vivo* typically spans several years, trauma-induced changes in cartilage following *in vitro* injury can occur over a much shorter time frame, ranging from hours to days. This highlights the acute nature of the mechanical injury responses *in vitro*, which can serve as a model for studying the early stages of OA development under mechanical stress.

Chondrocytes, the main cell type in cartilage, sense and respond to mechanical loading, notably through integrin–matrix interactions, activation of mechanosensitive ion channels such as activation of transient receptor potential vanilloid (TRPV) channels or piezo-channels, and primary cilium-induced signaling.^16^ Although physiological mechanical constraints on the joint help maintain tissue homeostasis, joint overloading favors pathological outcomes including catabolism and therefore cartilage degeneration. Indeed, depending on the loading magnitude, chondrocytes can exhibit responses ranging from anabolism to catabolism.^17^ Chondrocyte mechanical loading is known to induce signaling pathways, such as the MAPK/ERK pathways, capable of modulating cell metabolism or favoring growth factor release.^18–21^ Mesenchymal stromal cells (MSCs) can also sense mechanical stimulations (MS), notably through TRPV4, which affect ECM homeostasis and composition.^22,23^ In summary, although moderate compression can enhance ECM properties, excessive mechanical loading is linked with the development of osteoarthritis features, making it relevant for developing appropriate models to study OA.

Animal models, including spontaneous and induced OA in small and large animals, are valuable for developing treatments against OA.^24^ Small animal models are cost-effective but fail to replicate human joint anatomy, whereas large animal models, like horses, have anatomical similarities but are costly to manage.^25^ The horse model is particularly useful for studying OA and improving treatments for both horses and humans. In line with the 3Rs principles (refine, reduce, replace), *in vitro* OA models have been developed.^26^ While monolayer models do not recapitulate the articular cartilage architecture, 3D cartilage models with chondrocytes or MSCs seem more appropriate.^24,27–30^ Articular cartilage models can also be cocultured with OA synovium or incubated with pro-inflammatory cytokines to simulate OA. However, coculture requires biopsies and raises ethical issues, while cytokines like interleukin-1 beta (IL-1β) do not replicate major OA characteristics, such as HTRA1 upregulation.^31,32^ Developing relevant models is essential to assess the therapeutic potential of emerging treatments in OA.

In this study, we aimed to develop an equine 3D articular cartilage model exhibiting osteoarthritic features. We synthesized a hyaline-like cartilage model from equine BM-MSCs in collagen sponges cultured in chondrogenic medium for 21 days. Subsequently, we applied compressive forces to the MSC-derived cartilage and evaluated the ability of the cells to sense MS as well as the effects of MS on cell viability, ECM composition, and articular cartilage markers expression.

## 2 Materials and Methods

### 2.1 Cell Isolation and Culture

BM-MSCs were previously isolated from horses with ages ranging from 3 to 12 years old, characterized, and biobanked in liquid nitrogen.^27,33^ Isolated MSCs were previously characterized through immunophenotyping, confirming the presence of CD29, CD44, CD73, and CD90 and the absence of CD45 and type II major histocompatibility complex (MHC).^27,33^ Additionally, their multipotential and proliferative capacities were previously assessed. Characterized MSCs were defrosted, seeded at 5000 cells/cm^2^, and amplified in LG-DMEM (Eurobio Scientific) supplemented with 20% fetal bovine serum (FBS; Eurobio Scientific) and 1% penicillin–streptomycin–amphotericin B (PSA; Eurobio Scientific) at 37 °C in a 5% CO_2_-humidified atmosphere.

Equine articular chondrocytes (eAC) were isolated from the cartilage of carpal and femoral condyles of horses with ages ranging from 3 to 10 years old, as previously described.^28^

### 2.2 Chondrogenic Differentiation in 3D

MSCs at P3 were trypsinized and seeded into type I/III collagen sponges (5 mm in diameter, 2 mm in thickness; Symatèse Biomateriaux, France) at 5 × 10^5^ cells/sponge in 20 μL of Incomplete Chondrogenic Medium (ICM, composed of HG-DMEM (Eurobio Scientific) with 10^–7^ M dexamethasone (Sigma-Aldrich), 50 μg/mL ascorbic acid-2-phosphate (Sigma-Aldrich), 40 μg/mL l-proline (Sigma-Aldrich), 1 nM sodium pyruvate (Gibco, Thermo Fisher Scientific), 1% Insulin–Transferrin–Selenium (ITS; Gibco), as previously described.^33^ After 1 h of incubation, the seeded sponges were transferred in 24-well plates and incubated with ICM supplemented with 50 ng/mL recombinant human bone morphogenetic factor 2 (BMP2; Inductos, 12 mg dibotermin alpha, Medtronic France, Paris, France) and 10 ng/mL transforming growth factor beta 1 (TGF-β1, Miltenyi Biotec, Bergisch Gladbach, Germany). This chondrogenic medium was changed twice a week for 21 days, which is required to generate our cartilage-like model. The 3D cultures were maintained at 37 °C in a humidified atmosphere with 5% CO_2_.

### 2.3 Mechanical Stimulation (MS) of the Cartilage-Like *In Vitro* Model

Dynamic compression was performed using the Flexcell FX-5000C Compression System (Flexcell International Corporation, Burlington, Vermont, USA) based on the protocols previously described^18,34,35^ with some modifications. After 21 days of culture, cartilage-like *in vitro* models were placed in each well of a BioPress compression culture six-well plate with 4 mL of chondrogenic medium. A stationary platen was then positioned on top of the wells, and the entire six-well plate was inserted into the BioPress clamping system. Cartilage-like *in vitro* models were subjected to sinusoidal dynamic compression. The compression regime was set to generate pulses of 2.5 kPa at a frequency of 0.2 Hz for 15 min, which corresponds to a theoretical pressure of 110 kPa applied to the cartilage-like *in vitro* model. This compression regime was designed to impose a greater mechanical constraint than those described in previous studies.^18,35^ This regime was applied either once or every 36 h until day 24 (2 cycles of MS) or day 28 (5 cycles of MS). Control cartilage-like *in vitro* models were not subjected to compression and were cultured under the same temperature and duration.

### 2.4 Western Blots

Compressed and uncompressed cartilage-like *in vitro* models were rinsed once with ice-cold PBS and freezed in liquid nitrogen before storage at −80 °C. Sponges were crushed, and total proteins were extracted at 4 °C using RIPA-lysis buffer supplemented with protease and phosphatase inhibitors as previously described.^36^ The Bradford assay was used to assess the protein concentration (Bio-Rad, Hercules, California, USA). A 10 μg amount of total protein was resolved on 10% polyacrylamide gels with 0.1% SDS and subsequently transferred to a polyvinylidene difluoride membrane (PVDF; Bio-Rad) using the Trans-Blot Turbo Transfer system (Bio-Rad). The membranes were incubated with 10% nonfat milk powder in Tris-buffered saline with 0.1% Tween (TBS-T) for 1 h to block nonspecific binding sites. After washes in TBS-T, membranes were incubated overnight at 4 °C with primary antibodies listed in Table 1.

**Table 1:** Antibodies Used for the Western Blot Analysis of Target Proteins

**target protein**	**type**	**dilution**	**supplier**
type I collagen	rabbit polyclonal	1:3000	Novotec, Bron, France
GAPDH	mouse monoclonal	1:3000	Santa Cruz Biotechnology, Dallas, Texas, USA
PCNA	mouse monoclonal	1:1000	Santa Cruz Biotechnology, Dallas, Texas, USA
HtrA1	rabbit polyclonal	1:1000	Abcepta, San Diego, California, USA
type IIB collagen	rabbit polyclonal	1:1000	Covalab, Villeurbanne, France
type X collagen	mouse monoclonal	1:1000	Sigma, Saint Louis, Missouri, USA
Phospo-p44/42 MAPK	rabbit polyclonal	1:1000	Cell Signaling, Danvers, Massachusetts, USA
p44/42 MAPK	rabbit polyclonal	1:1000	Cell Signaling, Danvers, Massachusetts, USA
antirabbit antibody, HRP-conjugated	goat polyclonal	1:5000	Jackson Immunoresearch, West Grove, Pennsylvania, USA
antimouse antibody, HRP-conjugated	goat polyclonal	1:5000	Jackson Immunoresearch, West Grove, Pennsylvania, USA

The following day, membranes were washed in TBS-T, followed by an incubation with HRP-conjugated goat antirabbit or mouse IgG antibodies listed in Table 1. The ChemiDoc Touch Imaging System (Bio-Rad) was used to visualize the chemiluminescence (Clarity Western ECL Substrate; Bio-Rad).

### 2.5 RNA Extraction and RT-qPCR

Total RNA was extracted using TRIzol Reagent (Invitrogen Life Technologies), and 1 μg of DNase-treated total RNA was reversed-transcribed into cDNA using the iScript Reverse Transcription Supermix (Bio-Rad) according to the manufacturer’s instructions. Real-time PCR was performed using a GoTaq Probe qPCR Master Mix (Promega, Charbonnières, France) on a CFX96 Touch (Bio-Rad). Primers used are listed in Table 2.

**Table 2:** Primer Sequences Used for the Analysis of Gene Expression

**target gene**	**forward sequence**	**reverse sequence**
*ACAN*	TGTCAACAACAATGCCCAAGAC	CTTCTTCCGCCCAAAGGTCC
β-*ACTIN*	GATGATGATATCGCCGCGCTC	TGCCCCACGTATGAGTCCTT
*ADAMTS5*	AAGGGACACCATGTGGCAAA	CCCACATGAGCGAGAACACT
*COL1A1*	TGCCGTGACCTCAAGATGTG	CGTCTCCATGTTGCAGAAGA
*COL2A1*	GGCAATAGCAGGTTCACGTACA	CGATAACAGTCTTGCCCCACTT
*COL10A1*	GCACCCCAGTAATGTACACCTATG	GAGCCACACCTGGTCATTTTC
*COMP*	ATCCGAAATGCGGTGGACAA	TCCTTGTCTTGGTCGCTGTC
*PPIA*	CCCTACCGTGTTCTTCGACA	GTGAAGTACACCACCCTGACA
*PRG4*	CTACCACCCAACGAACAAA	ACTGTTGTCTCCTTATTGGGTGT
*RUNX2*	GCAGTTCCCAAGCATTTCAT	CACTCTGGCTTTGGGAAGAG
*SOX9*	CAAGAAGGACCACCCGGACTA	GGAGATGTGTGTCTGCTCCGT
*TRPV4*	CCGCGACATCTACTACCGAG	AGGGGCAGCTCACCAAAGTA

Relative gene expression of triplicate samples was analyzed using the Normalized Expression mode (ΔΔCq) available in the Bio-Rad CFX Manager software using two housekeeping genes, β*-ACTIN* and *PPIA*. mRNA extracted from Equine Articular Chondrocytes (eAC-P0) was used as a control for RT-qPCR.

### 2.6 Cell Proliferation and TUNEL Assays

Cell proliferation in compressed and uncompressed cartilage-like *in vitro* models was evaluated at day 24 using the thymidine analogue EdU, which can be incorporated into replicating DNA (EdU Cell Proliferation Image Kit, Abbkine, Atlanta, Georgia, USA). At the beginning of mechanical stimulation (day 21), 10 μM EdU was added to the cartilage-like *in vitro* models for 3 days. On day 24, cartilage-like *in vitro* models were harvested, washed once in preheated PBS to remove unincorporated EdU, and fixed in 10% formalin (Sigma-Aldrich) at room temperature for 16 h.

Cartilage-like *in vitro* models were embedded in paraffin with an automaton (VIRTUAL’HIS, Université Caen Normandie, France). Then, 6 μm sections were made with a microtome and mounted on silanized slides. The sections were deparaffinized by two successive baths of xylene for 5 min each, followed by rehydration in 100% ethanol (two baths of 5 min), 90% ethanol (5 min), 70% ethanol (5 min), and finally distilled water. The deparaffinized sections were then permeabilized with 0.5% Triton X-100 for 20 min, followed by two washes with bovine serum albumin (BSA) wash solution, and incubated with 100 μL of Click-iT reaction mixture for 30 min at room temperature in the dark. After washing, a DAPI solution was applied for nuclear staining.

Cell apoptosis was evaluated on compressed and uncompressed paraffin-embedded cartilage-like *in vitro* models, at days 24 and 28, using the One-step TUNEL Assay Kit (MyBioSource, San Diego, California, USA) according to the manufacturer’s instructions. Briefly, the 6 μm deparaffinized slides were incubated with a proteinase K working solution (37 °C for 20 min), washed with PBS, incubated with a working solution of TdT and labeled dUTP (37 °C for 90 min), washed again, incubated with a DAPI working solution (room temperature for 5 min), and washed before mounting with a FluoreGuard Mounting Medium (ScyTek Laboratories, Inc., Logan, Utah, USA). Positive and negative controls (prior incubation with Dnase and absence of TdT enzyme, respectively) were also performed.

EdU-positive cells, fluorescein-dUTP labeling cells, and DAPI-stained nuclei were visualized with an Olympus VS 120-L100-123 fluorescence scanner and counted with the QuPath 0.5.0 software and StarDist detection method.^37,38^

### 2.7 Histochemical and Immunohistochemical Analyses

After 21, 24, or 28 days of chondrogenic differentiation with or without MS, cartilage-like *in vitro* models were harvested, fixed in 10% formalin, embedded in paraffin, and cut into 6 μm paraffin sections as described above. The deparaffinized sections were stained with hematoxylin–eosin–saffron (HES; Labonord S.A., VWR International, Templemars, France), Alcian blue (1%, pH 2.5; Sigma-Aldrich), and Alizarin red S (2%, pH 4.1; Sigma-Aldrich) according to routine protocols. Immunostaining was initiated by a pretreatment of 30 min with 0.5% hyaluronidase in PBS containing 3% of BSA, followed by permeabilization with PBS containing 0.2% of Tween 20 and inhibition of the endogenous peroxidases with Dako real peroxidase blocking solution (DAKO, Agilent Technologies, Inc., Santa Clara, California, USA). Sections were then incubated overnight with rabbit antihuman type II collagen (1:250 dilution; Novotec, Bron, France). An HRP-conjugated antirabbit secondary antibody (EnVision+; DAKO) was applied to each section. Signals were revealed using diaminobenzidine tetrahydrochloride (DAB; DAKO) as a chromogen. Mayer’s hematoxylin (DAKO) and an ammonium hydroxide solution (0.037 M; Honeywell Fluka, Thermo Fisher Scientific) were used for counterstaining. The primary antibodies were omitted for the negative controls. All slides were mounted with Eukitt (DAKO) after dehydration.

An Olympus VS 120-L100-123 scanner was used to digitalize the histological slides. Images were analyzed with QuPath 0.5.0 software.

### 2.8 Cartilage Oligomeric Matrix Protein ELISA

Horse Cartilage Oligomeric Matrix Protein (COMP) Bioassay ELISA kit (USBiological Life Sciences, Salem, Massachusetts, USA) was used to evaluate the COMP released in the culture media of compressed and uncompressed cartilage-like *in vitro* models at days 24 and 28. COMP levels were measured in 100 μL of media, following the manufacturer recommendations. Each sample was quantified in duplicate. Absorbances were read at 450 nm on a microplate reader (Spark 10M, TECAN, Lyon, France). The concentrations were calculated in ng/mL using a standard curve. The detection range of the COMP kit is 1.25–80 ng/mL.

### 2.9 GAG Assay

The total amount of glycosaminoglycans (GAGs) including sulfated and nonsulfated GAGs was evaluated on the culture media after 21, 24, or 28 days of chondrogenic differentiation with or without MS, using the total GAG assay kit (Abcam; Cambridge, UK). In this colorimetric assay, GAGs interact with a specific probe to form a colored product, which is measured by absorbance at 400 nm. Briefly, 10 μL of GAG assay buffer and 170 μL of GAG probe were mixed with 90 μL of culture media and incubated for 2 min at room temperature. Each sample was quantified in duplicate. Absorbances were measured with a microplate reader (Spark 10M, TECAN). The amount of GAG was evaluated from a standard curve of GAG after subtraction of the sample blank and expressed in μg/mL. The limit of detection of this kit is 1 μg of chondroitin sulfate or hyaluronic acid.

### 2.10 Nitrite Assay

Nitrite levels were measured in the culture media after 24 or 28 days of chondrogenic differentiation with or without MS, using the Griess Reagent Kit for nitrite determination (Molecular Probes, Eugene, Oregon, USA). Briefly, 120 μL of medium in duplicate was used for nitrite determination (μM) in accordance with the manufacturer’s instructions, as previously described.^31^ The detection limit of this kit is 1 μM.

### 2.11 Cytokine Dosage

The MILLIPLEX Equine Cytokine/Chemokine Magnetic Bead Panel assay (Millipore, Merck KGaA, Darmstadt, Germany) was used to determine the concentration of 23 cytokines: chemokine (C–X–C motif) ligand 1 (CXCL1, GRO1), chemokine (CC-motif) ligand 2 (CCL2, MCP-1), CX3CL1 (Fractalkine), eotaxin (CCL11), fibroblast growth factor 2 (FGF-2), granulocyte colony-forming stimulating factor (G-CSF), granulocyte-macrophage (GM)-CSF, interferon-γ (IFN-γ), IFN-γ-induced protein 10 (IP-10, CXCL10), interleulin-1 alpha (IL-1α), IL-1β, IL-2, IL-4, IL-5, IL-6, IL-8 (CXCL8), IL-10, IL-12 (p70), IL-13, IL-17A, IL-18, TNF-α, and regulated upon activation normal T cell expressed and secreted (RANTES, CCL5). The detection threshold was specific for each cytokine. The quantification of these cytokines was performed in duplicate using 25 μL of culture media of compressed and uncompressed cartilage-like *in vitro* models at day 24 following the manufacturer’s guidelines, as previously described.^39^ Measurements were performed with the Luminex MAGPIX CCD imager (Luminex Corp., Texas, United States) and processed with Luminex xPONENT software.

### 2.12 Cytotoxicity Assay

A bioluminescence cytotoxicity assay kit (Interchim, Montluçon, France) was used to evaluate the cell death after 21, 24, or 28 days of chondrogenic differentiation with or without MS, as previously described.^32^ After MS, 80 μL of culture media was transferred in duplicate to a 96-well plate and incubated with 100 μL of assay reagent for 5 min at room temperature. A death control, corresponding to 100% cell death, was realized with an incubation of cartilage-like *in vitro* models for 15 min with 1% Triton X-100 (Sigma-Aldrich). The bioluminescence was read on a microplate reader (Spark Control Magellan, TECAN) and expressed as a percentage relative to the death control (100%).

### 2.13 MMP Activity Assay

Matrix metalloproteinase (MMP) activity was assessed using the Amplite Universal Fluorimetric MMP Activity Assay Kit (AAT Bioquest, #13510, Sunnyvale, California, USA) in the supernatants of control and compressed samples following the kit instructions. 25 μL of each test sample was added to a solid black 96-well plate, along with 25 μL of the supplied assay buffer. Then, 50 μL of the MMP Green working solution was added to each well. After 30 min of incubation at 37 °C (protected from light), the plate was mixed, and fluorescence intensity was measured at Ex/Em = 485/535 nm (TECAN, Microplate Reader Spark).

### 2.14 Statistical Analyses

Statistical analyses were conducted using GraphPad Prism 8.0.1 software (San Diego, California, USA). The Shapiro–Wilk test was used to determine whether the data followed a Gaussian distribution. The significance of the results between the two groups was tested using the Mann–Whitney test. Differences were deemed significant at *p* ≤ 0.05.

## 3 Results

### 3.1 Equine BM-MSC-Derived Chondrocytes Express TRPV4 and Sense Mechanical Stimulations

First, we sought to determine whether equine BM-MSC-derived chondrocytes expressed mechanosensitive ion channel *TRPV4*. To that aim, we produced an MSC-derived cartilage-like *in vitro* model, as previously described.^27^ Briefly, equine BM-MSCs were seeded in collagen sponges and cultured for 21 days in a chondrogenic medium to produce cartilage-like *in vitro* models (Figure 1A).

**Figure 1:**
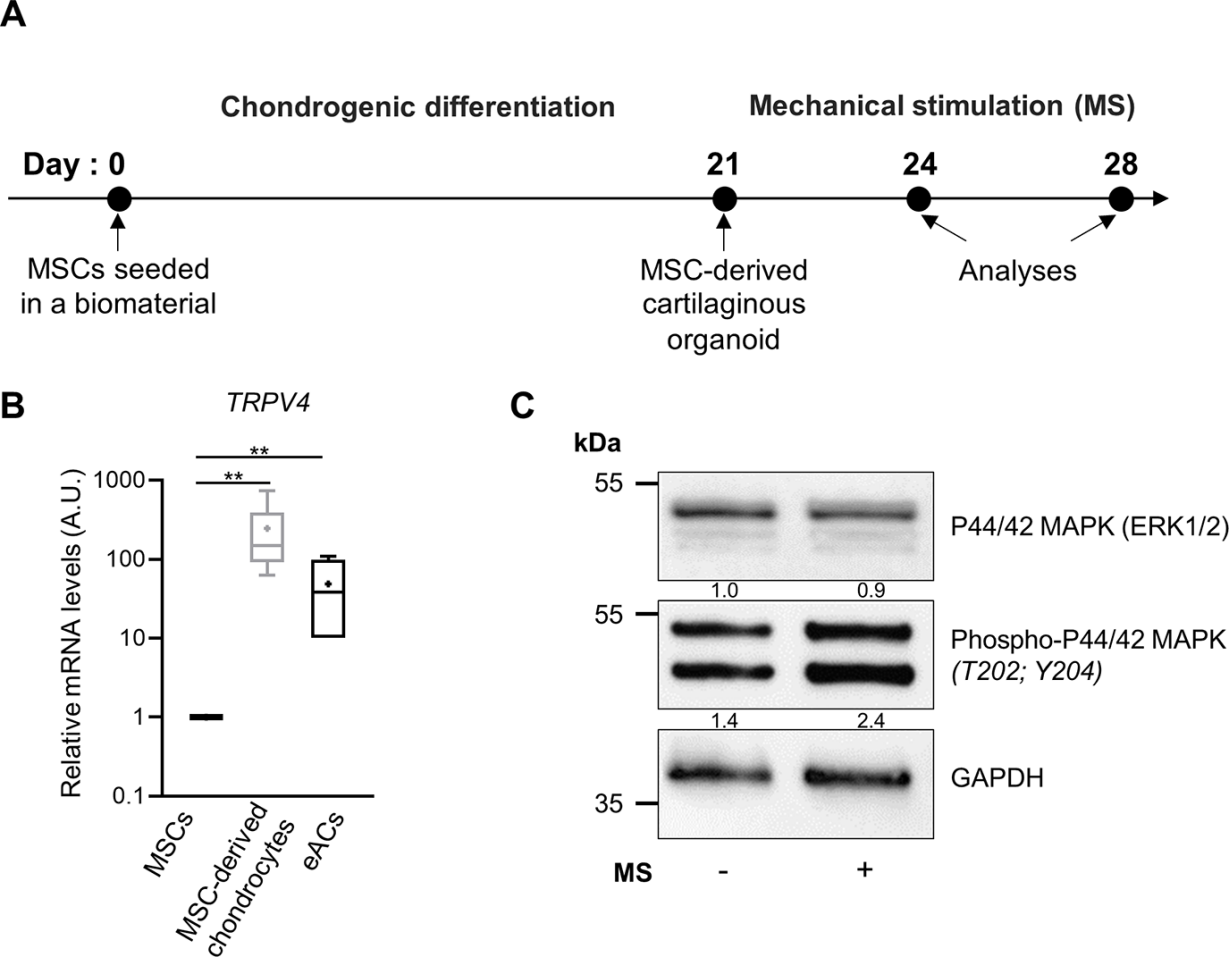
Equine BM-MSC-derived chondrocytes express TRPV4 and sense mechanical stimulations. (A) Scheme of the compression system and experimental timeline. BM-MSCs were seeded at passage 4 in collagen sponges (5 × 10^5^ cells/sponge) and cultured for 21 days in a chondrogenic medium to produce a cartilage-like *in vitro* model. (B) Box plot of the relative mRNA levels of *TRPV4* (mean (cross), median (line), and ±SD error bars, *n* = 6). Total mRNA was extracted, and RT-qPCRs were performed to assess the transcript level of target genes, as shown in the panel. *β-ACTIN* and *PPIA* were used as reference genes. The Mann–Whitney test was used to analyze differences compared to MSCs (**p > 0.01). (C) Representative immunoblots of the activation of the P44/42 MAPK pathway in 21-day cartilage-like *in vitro* models cultured in static condition (−) or subjected to mechanical stimulation (MS +) (15 min of compression under 2.5 kPa at 0.2 Hz) (*n* = 4). The densitometric analysis of each band is relative to GAPDH and is indicated below the blots. BMP2: bone morphogenetic protein 2; TGFβ1: transforming growth factor β-1.

The transcript levels of *TRPV4* were compared among MSCs, MSC-derived chondrocytes, and chondrocytes isolated from horse articular cartilage. Interestingly, the differentiation of MSCs into chondrocytes induced a 100-fold increase in *TRPV4* levels, reaching values approaching those observed in eACs (Figure 1B). Then, to confirm MSC-derived chondrocytes can sense mechanical stimulations (MS), the cartilage-like *in vitro* models were cultured under static conditions or subjected to MS for 15 min at 2.5 kPa and 0.2 Hz (Figure 1A). As the P44/42 MAPK pathway can be activated by MS, the phosphorylation of P44/42 MAPK was analyzed. As expected, we observed an increase in the phosphorylation of P44/42 MAPK under MS compared to that under the static condition (Figure 1C). These findings indicate that MSC-derived chondrocytes express mechanosensitive ion *TRPV4* channels and can integrate MS into intracellular signals.

### 3.2 Mechanical Stimulations Decrease Equine BM-MSC-Derived Chondrocyte Proliferation

Knowing that BM-MSC-derived chondrocytes can sense MS, the next step was to investigate whether compression affects the cell viability. For this purpose, equine MSC-derived chondrocytes were incubated with EdU, a thymidine analogue that is incorporated into proliferating cells. Surprisingly, the number of EdU-positive cells decreased after 3 days under MS (D24), compared to the static condition (Figure 2A,B).

**Figure 2:**
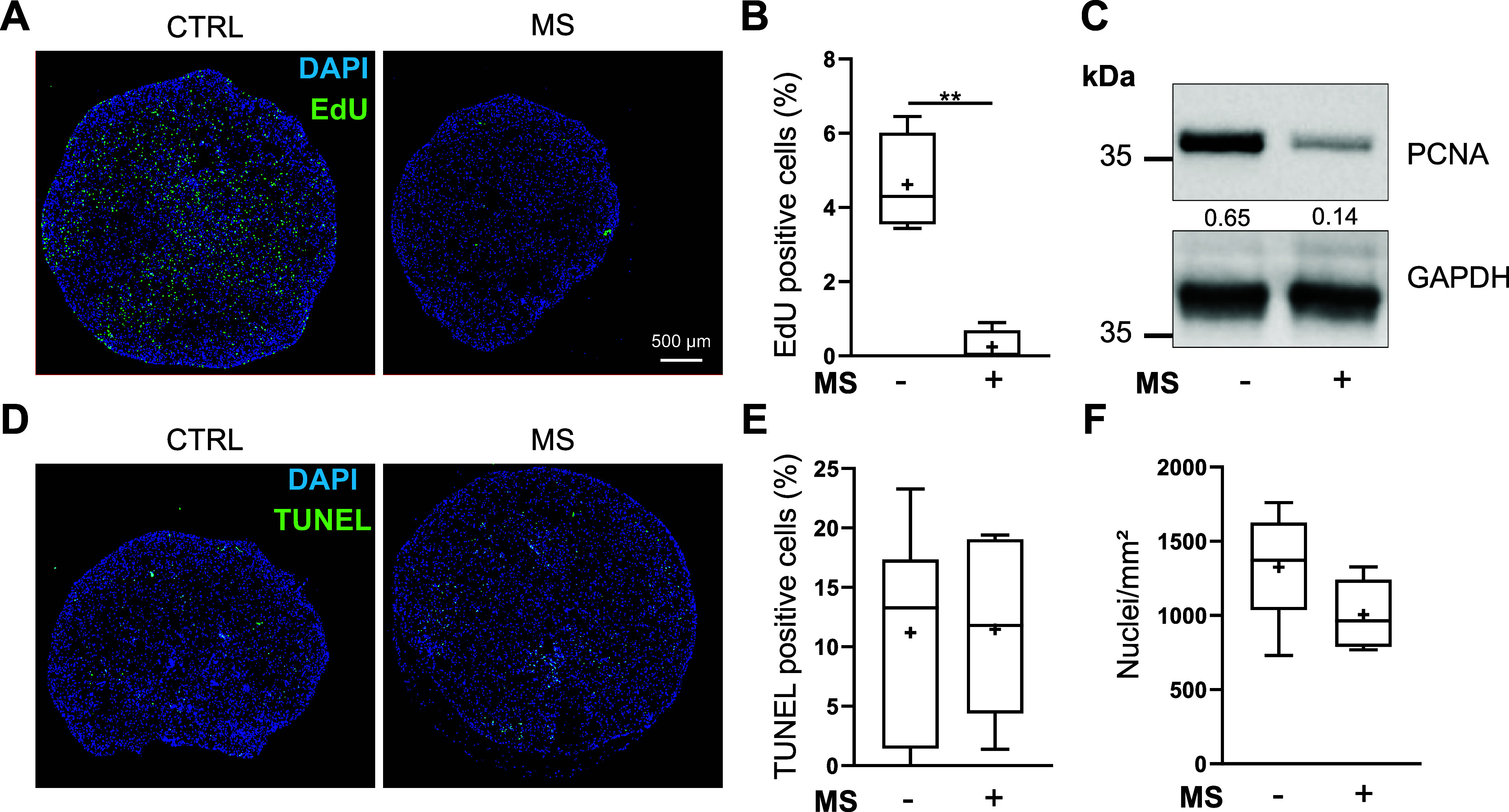
Mechanical stimulations decrease equine BM-MSC-derived chondrocyte proliferation. BM-MSCs were seeded at passage 4 in collagen sponges (5 × 10^5^ cells/sponge) and cultured for 21 days in a chondrogenic medium to produce a cartilage-like *in vitro* model. The cartilage-like *in vitro* models were then cultured in static condition (−) or subjected to mechanical stimulations (MS + for 3 days (15 min of compression under 2.5 kPa at 0.2 Hz every 36 h). The cartilage-like *in vitro* models were fixed in formalin overnight and embedded in paraffin, and the EdU (A) or the TUNEL (D) assays were performed (*n* = 6). (C) Representative immunoblots of the PCNA protein. The densitometric analysis of each band is relative to GAPDH and is indicated below the blot. (B) Box plot of the number of EdU, TUNEL-positive cells (E) and DAPI-stained nuclei (F) (mean (cross), median (line), and ±SD error bars, *n* = 6). The Mann–Whitney test was used to analyze differences compared to the cartilage-like *in vitro* model culture in static condition (CTRL) (**p > 0.01).

Similarly, the proliferating cell nuclear antigen (PCNA) protein amount decreased compared to that of the control condition (Figure 2C). Hence, the number of nuclei/mm^2^ was slightly decreased after 3 and 7 days of MS (Figure 2F and Supplementary Figure 1C). In parallel, to evaluate the impact on apoptosis, a TUNEL assay was performed. The proportion of apoptotic nuclei remained low either in control conditions or under MS, for 3 (D24) or 7 days (D28) (Figure 2D,E and Supplementary Figure 1A,B). Moreover, MS, regardless of the duration, did not induce cytotoxicity, measured by the release of adenylate kinase by damaged cells into the medium (Supplementary Figure 1D). These findings indicate that MS on equine MSC-derived chondrocytes reduces cell proliferation without affecting cell death or apoptosis.

### 3.3 Mechanical Stimulations Increase the mRNA Levels of Cartilage Typical Markers but Impair the Accumulation of ECM Components

To continue, we sought to identify whether MS is relevant to induce osteoarthritic features in cartilage-like *in vitro* models. Following MS, we investigated the mRNA levels of genes coding ECM components and key transcription factors as well as the quality of the ECM in the cartilage-like *in vitro* models. First, cartilage-like *in vitro* models cultured under static conditions did not exhibit statistically different levels of *ACAN* (aggrecan), *COL2A1* (alpha-1 chain of type II collagen), *SOX9* (SRY (sex-determining region Y)-box 9), *PRG4* (proteoglycan 4), *COL1A1* (alpha-1 chain of type I collagen), and *RUNX2* (Runt-related transcription factor 2) mRNA between D21 and D24/D28 (Figure 3). Hence, the *COL2A1/COL1A1* ratio was unchanged between D21 and D24/D28. On the contrary, the mRNA levels of *COMP* were decreased at D24. Regarding the mRNA levels of cartilage-like *in vitro* models that underwent MS, *COL2A1*, *SOX9*, *PRG4*, and *RUNX2* were increased whereas *COL1A1* remained unchanged, compared to D21. The *COL2A1, ACAN*, and *PRG4* mRNA levels even reach those observed in eACs. Thus, the *COL2A1*, *SOX9*, *PRG4*, and *RUNX2* mRNA levels and the *COL2A1/COL1A1 ratio* were higher when the cartilage-like *in vitro* models underwent MS (D24), compared to the cartilage-like *in vitro* model cultured under static conditions. A similar trend was observed after 7 days of MS (D28), except the *COL2A1/COL1A1* ratio, which decreased between D24 and D28 to reach a similar level than in static conditions, well below the eACs. The *COMP* levels were similar; either the cartilage-like *in vitro* models were cultured under static or MS conditions but remained higher than those observed in eACs.

**Figure 3:**
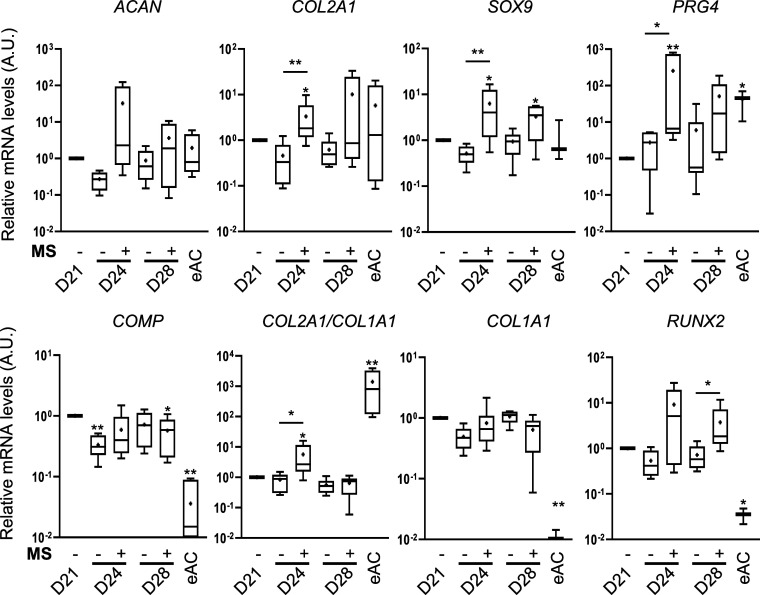
Mechanical stimulations increase mRNA levels of cartilage typical molecules. BM-MSCs were seeded at passage 4 in collagen sponges (5 × 10^5^ cells/sponge) and cultured for 21 days in a chondrogenic medium to produce a cartilage-like *in vitro* model. The cartilage-like *in vitro* models were then cultured in static condition (−) or subjected to mechanical stimulations (MS +) for 3 days (D24) or 7 days (D28) (15 min of compression under 2.5 kPa at 0.2 Hz every 36 h). (A) Box plot of the relative mRNA levels of cartilage typical molecules (mean (cross), median (line), ±SD error bars, *n* = 6). Total mRNA was extracted, and RT-qPCRs were performed to assess the transcript level of target genes as shown in the panel. *β-ACTIN* and *PPIA* were used as reference genes. The Mann–Whitney test was used to analyze differences compared to D21 and to the corresponding static condition (*p < 0.05; **p > 0.01).

Then, to investigate the quality of the ECM in the cartilage-like *in vitro* models, we assessed the levels of collagen proteins using Western blot analysis. Contrary to what *COL2A1* mRNA levels could have suggested, after 3 days of compression (D24), the amounts of type I collagen and type IIB collagen were lower in the MS condition compared to the static condition (Figure 4A).

**Figure 4:**
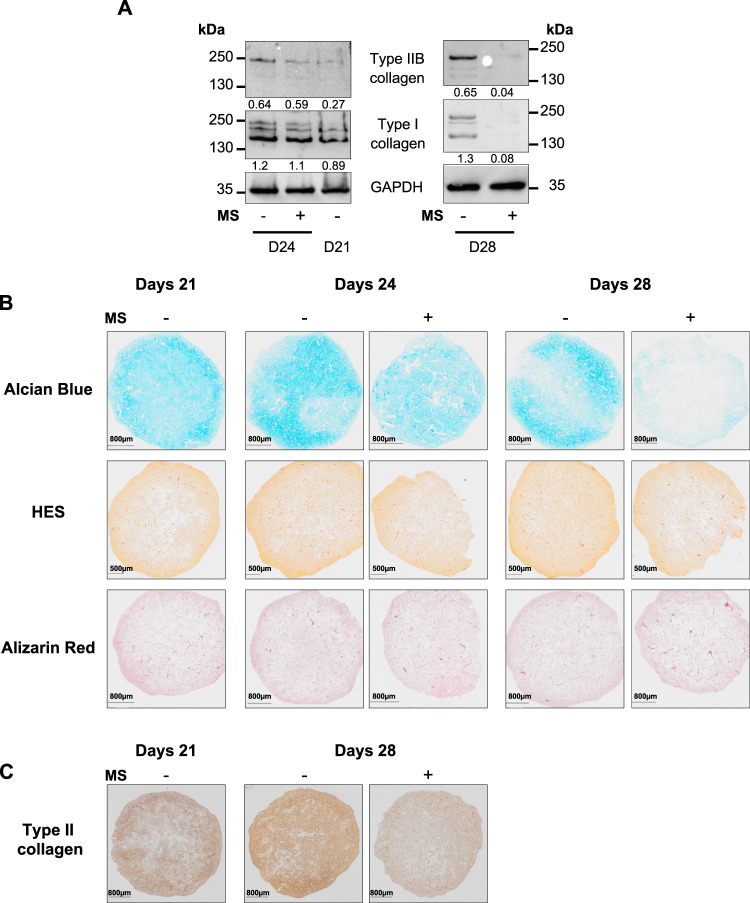
Mechanical stimulations impair the accumulation of ECM components. BM-MSCs were seeded at passage 4 in collagen sponges (5 × 10^5^ cells/sponge) and cultured for 21 days in a chondrogenic medium to produce a cartilage-like *in vitro* model. The cartilage-like *in vitro* models were then cultured in static condition (−) or subjected to mechanical stimulations (MS +) for 3 days (D24) or 7 days (D28) (15 min of compression under 2.5 kPa at 0.2 Hz every 36 h). (A) Representative immunoblots of collagens and GAPDH proteins (*n* = 6). The densitometric analysis of each band is relative to GAPDH and is indicated below the blots. (B) Alcian Blue, HES, and Alizarin Red S staining microphotographs of cartilage-like *in vitro* models cultured in static condition (−) or subjected to mechanical stimulations (+) (*n* = 6). (C) Immunostaining of type II collagen: microphotographs of cartilage-like *in vitro* models cultured in static condition (−) or subjected to mechanical stimulations (+) (*n* = 4).

Furthermore, after 7 days of MS (D28), there was a drastic decrease in the amounts of type I and type IIB collagens relative to the static conditions (Figure 4A). Hence, MS of cartilage-like *in vitro* models leads to an increase in the mRNA levels of some ECM components, which are not reflected at the protein level. Moreover, MS does not appear to promote cartilage hypertrophy. Although *COL10A1* expression may show a slight increase at the mRNA level, this trend is not reflected at the protein level (Supplementary Figure 2).

Finally, we performed histological analyses of the cartilage-like *in vitro* models to visualize the ECM using different staining methods: Alcian Blue to detect glycosaminoglycans (GAGs), Alizarin Red to detect calcium deposits, and hematoxylin–eosin–saffron (HES) to stain cell nuclei in blue, cytoplasm in pink, and collagen fibers in orange. At 21 days, the cartilage-like *in vitro* models were filled with neo-synthesized ECM, heterogeneously distributed throughout the sponge (Figure 4B). Indeed, a higher intensity of staining was observed at the periphery compared with the center. Regardless of the culture condition, cartilage-like *in vitro* models did not exhibit obvious signs of ossification, since the Alizarin Red staining remained weak. After 3 days of MS (D24), we observed a decrease in GAG and collagen amounts, compared to the cartilage-like *in vitro* models cultured under static conditions at D21 and D24. After 7 days of compression (D28), the reduction in ECM amount became more pronounced, notably regarding the type II collagen (Figure 4B,C).

These findings suggest that MS impairs the quality of the ECM by reducing the GAG and collagen protein amounts in the cartilage-like *in vitro* models without decreasing the mRNA level of major ECM component.

### 3.4 Mechanical Stimulations of Equine BM-MSC-Derived Chondrocytes Promote Catabolic-Associated Events

We wondered whether an increase in catabolism could trigger the ECM alteration upon MS. Pro-inflammatory cytokines play an important role in the induction of catabolic events. Out of the 23 cytokines tested, only three reached detection thresholds. The levels of fibroblast growth factor 2 (FGF-2), granulocyte colony-stimulating factor (G-CSF), and granulocyte-macrophage colony-stimulating factor (GM-CSF) were below detection thresholds under static conditions. However, under mechanical compression, the concentrations of these three cytokines increased significantly (Table 3), indicating that MS induced an increase in either the release or the expression of FGF-2, G-CSF, and GM-CSF.

**Table 3:** Impact of Mechanical Stimulation on Cytokine Release^a^

**target**	**control (pg/mL)**	**MS (pg/mL)**
FGF-2	N/A	51.96 ± 33.42
G-CSF	N/A	162.41 ± 60.98
GM-CSF	N/A	15.875 ± 7.08

a FGF-2, G-CSF, and GM-CSF concentrations were measured in the media of cartilage-like *in vitro* models cultured in static condition (control) or under MS for 3 days (D24) (compression: 2.5 kPa, 0.2 Hz, 15 min/36h) (*n* = 4).

Then, the mRNA levels of *ADAMTS5,* a major aggrecanase involved in proteoglycan degradation during OA, increased approximately 50-fold after 3 days of MS (D24), and this trend appeared to continue after 7 days (D28) (Figure 5A).

**Figure 5:**
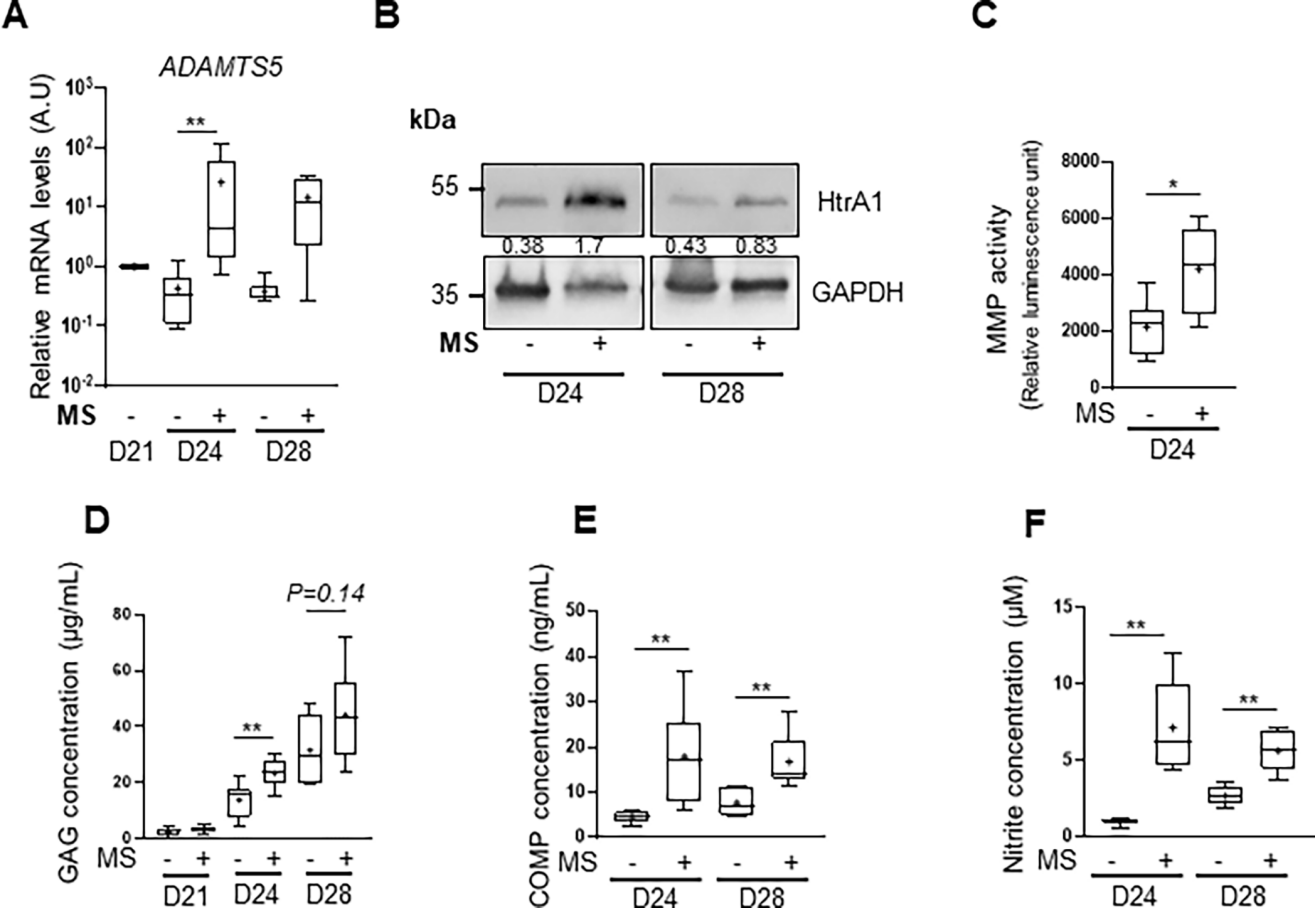
Mechanical stimulations of equine BM-MSC-derived chondrocytes promote catabolic-associated events. BM-MSCs were seeded at passage 4 in collagen sponges (5 × 10^5^ cells/sponge) and cultured for 21 days in a chondrogenic medium to produce a cartilage-like *in vitro* model. The cartilage-like *in vitro* models were then cultured in static condition (−) or subjected to mechanical stimulations (MS +) for 3 days (D24) or 7 days (D28) (15 min of compression under 2.5 kPa at 0.2 Hz every 36 h). (A) Box plot of the relative mRNA levels of *ADAMTS5* (mean (cross), median (line), ± SD error bars, *n* = 6). Total mRNA was extracted, and RT-qPCRs were performed to assess the transcript level of target genes, as shown in the panel. *β-ACTIN* and *PPIA* were used as reference genes. (B) Representative immunoblots of the HtrA1 and GAPDH proteins (*n* = 6). The densitometric analysis of each band is relative to GAPDH and is indicated below the blots. (C) Box plot of the GAG and nitrite (D) concentration measured in the media of cartilage-like *in vitro* models cultured in static condition (−) or under MS (+) (*n* = 6). (F) MMP activity was evaluated in the media of cartilage-like *in vitro* models cultured in static condition (−) or under MS (+) (*n* = 6). The Mann–Whitney test was used to analyze differences compared to the corresponding static condition (**p > 0.01).

Additionally, HtrA1, a serine protease known to play several roles in OA that converges toward a reduced and impaired ECM, was also investigated. The evaluation of HtrA1 protein levels revealed a raise after 3 days of MS, which persisted after 7 days (Figure 5B). Similarly, MMP activity was elevated following 3 days of MS (Figure 5C), supporting the involvement of matrix enzymes in the observed response. Given the high proteoglycan/GAG content of the cartilage-like *in vitro* model ECM (Figure 4B), its degradation would probably lead to its release into the medium. For this reason, we measured the levels of GAGs in the culture medium. The GAG concentrations seemed to increase over time under static conditions, from day 24 to day 28. One cycle of MS (D21) did not affect the GAG concentration in the medium (Figure 5D). On the contrary, MS induced an elevation in both GAG and COMP concentrations after 3 days (D24) and 7 days (D28) (Figure 5D,E). Finally, we measured the nitric oxide (NO), a key inflammatory mediator in OA. As nitric oxide (NO) is unstable in solution and rapidly transforms into nitrite, the concentration of nitrite was measured in the culture medium. We found that the nitrite concentration significantly increased after both 3 and 7 days of compression (Figure 5F). Altogether, these findings suggest that MS could increase inflammatory mediators and promote catabolism in equine BM-MSC-derived chondrocytes and therefore ECM degradation.

## 4 Discussion

In this study, we aimed to develop a 3D equine cartilage model exhibiting OA features by using MSCs. Human cartilage is subjected to a wide range of mechanical stresses *in vivo*, with hip joint loads reaching 5–8 MPa during walking. In contrast, impacts above 25 MPa can lead to significant chondrocyte death.^40^ Such loading conditions cannot be directly replicated *in vitro* as an *in vitro* cartilage model does not fully mimic native cartilage, particularly in terms of thickness, and lacks the supportive surrounding tissues of the joint. Nevertheless, based on previous studies using the same bioreactor system, we applied forces that are capable of approximating excessive mechanical stress on the model. Thus, we investigated how MSC-derived cartilage-like *in vitro* models respond to MS at pressures capable of inducing injurious stress, focusing on its effects on cell viability, ECM composition, and expression of key articular cartilage markers.

Given their size, a theoretical pressure of approximately 110 kPa was applied to equine cartilage-like *in vitro* models derived from BM-MSCs that had been differentiated for 21 days, as described previously.^27,29^ In this study, we first confirmed that equine BM-MSC-derived chondrocytes express mechanosensitive ion channel *TRPV4*, which is known to play a key role in mechanobiology. Additionally, the activation of the P44/42 MAPK pathway in response to MS further supports the capacity of MSC-derived chondrocytes to transduce mechanical signals into intracellular responses. These findings are consistent with previous studies emphasizing the importance of mechanosensitive pathways in cartilage biology.^18,22,41^

A significant finding here is that MS reduces the proliferation of MSC-derived chondrocytes, as evidenced by a decrease in EdU-positive cells, a lower PCNA protein amount, and a slight reduction in the number of nuclei. Importantly, this decrease in proliferation was not accompanied by increased cell death or apoptosis, as the TUNEL assay and adenylate kinase measurements showed low apoptosis and cytotoxicity levels. Thus, while mechanical loading may inhibit cell division in our model, it does not appear to trigger harmful effects on cell viability, which is in accordance with previous cartilage mechanobiology studies involving cartilage explants^42^ and MSC-derived models.^43^ Nevertheless, other studies have shown that once a certain threshold strain amplitude is exceeded, mechanical loading becomes harmful to cartilage,^44^ leading to either chondrocyte apoptosis^45^ or necrosis by direct mechanical change. Additionally, injurious compression on cartilage explants has been found to reduce cell viability as the strain rate increases.^13^ Therefore, the compression characteristics and the *in vitro* model are critical parameters to consider when studying the cartilage response to MS, particularly concerning cell viability and cell phenotype.

The results revealed the complex effects of MS on ECM composition. While mRNA levels of key cartilage ECM components such as *COL2A1*, *SOX9*, and *PRG4* increased under mechanical loading, protein amounts of collagen type IIB and type I, as well as GAG staining, decreased following MS. This discrepancy between mRNA and protein levels raises questions about the balance between ECM synthesis and degradation under mechanical stress. This suggests that post-transcriptional regulation may have occurred with the observed ECM downregulation possibly resulting from increased ECM catabolism rather than reduced synthesis. Histological analysis further reinforced this finding, showing a marked reduction in GAGs and, to a lesser extent, collagen content, key components of cartilage tissue. However, no signs of calcification were observed, attesting that MSC-derived chondrocytes did not acquire ossification features. These results suggest that mechanical loading stimulates both ECM remodeling and breakdown, key processes associated with the early stages of OA pathology.^8^

Given the reduced GAG and collagen content in the cartilage-like *in vitro* models, we hypothesized that mechanical loading might promote catabolic activity in our model. Our data showed that proteoglycan content in the ECM of the cartilage-like model decreased after 3 or 7 days of MS, which was accompanied by the release of GAGs into the supernatant and an increase in the expression of *ADAMTS5*, a key enzyme involved in aggrecan degradation during OA.^8^ Interestingly, HtrA1, a serine protease associated with OA and ECM impairment, along with MMP activity, was upregulated under MS. The upregulation of these proteases is consistent with the marked downregulation of collagen levels. These results are consistent with those obtained in other studies, where cells that remained viable after injury showed increased levels of mRNA for matrix-degrading enzymes,^46^ and pulse-chase radiolabeling experiments indicated that the matrix surrounding these viable cells may undergo accelerated degradation.^47^

In addition to these molecular changes, we observed under compression elevated levels of GAGs, COMP, and nitrite in the culture medium. This release of GAGs may be partially attributed to mechanical disruption of the matrix like previously demonstrated.^48,49^ Elevated COMP levels in the synovial fluid and serum are well established as being associated with OA, to the extent that some studies propose its use as a potential biomarker.^50,51^ Thus, the elevated levels of COMP observed in the supernatant of the cartilage-like *in vitro* model that underwent MS suggest that the cartilage-like *in vitro* model might have shifted to an osteoarthritic-like phenotype. Moreover, nitric oxide (NO, along with IL-1β, TNF-α, and prostaglandins, perpetuates cartilage inflammation and degradation. NO can also contribute to oxidative stress when it reacts with reactive oxygen species (ROS). Previous studies have shown that injurious loading of articular cartilage compromises chondrocyte respiratory function, leading to increased ROS formation, which mirrors the pathogenesis of OA.^52,53^ Mitochondrial ROS are key contributors to cartilage metabolic adaptation,^54^ and excessive ROS production is known to accelerate ECM degradation by reducing matrix biosynthesis, increasing cell death and proteolytic enzyme activity, altering cell–matrix interaction, and inhibiting TIMPs.^55–58^ Therefore, NO could play a pivotal role in initiating deleterious mechanisms following mechanical compression and could contribute to the previously reported changes in proteoglycan turnover caused by mechanical stress.^59^

Then, we sought to determine whether MS could have triggered the production or release of pro-inflammatory cytokines in the culture supernatant. MS led to a significant increase in the inflammatory mediators GM-CSF and G-CSF. GM-CSF has been identified as an important mediator in the progression of both pain and disease in an experimental OA model, in addition to its known role in rheumatoid arthritis.^60^ G-CSF whose levels rise markedly under stress plays a role in regulating the inflammatory response in models of inflammatory arthritis.^61^ However, the direct role of protease upregulation in MSC-derived chondrocytes and colony-stimulating factors (CSFs) still needs to be established. CSFs may form an important “CSF network” involving communication between stimulated macrophage, neutrophils, and neighboring cell type, such as chondrocytes, in inflammatory conditions. There is also evidence linking CSFs to pro-inflammatory cytokines, such as TNF and IL-1, which are secreted by macrophages and contribute to inflammation.^62^ However, numerous pro-inflammatory cytokines known to be upregulated in OA such as Il-1β, Il-6, and TNF-α were not detected. This result could be in part attributed to the fact that pro-inflammatory cytokines are synthesized by several cell types that compose and invade the diarthrodial joint during OA, notably including immune cells. Therefore, to obtain a comprehensive OA model, multi-tissue *in vitro* models will be needed; however, these models also have several limitations.

In this study, the application of MS appeared to influence the FGF-2 signaling axis, as evidenced by the increased levels of FGF-2 in the culture medium. FGF-2 has been shown to mediate the immediate response of articular cartilage to mechanical injury, inducing the synthesis of several chondrocyte proteins, including MMPs 1 and 3, TIMP-1, and glycoprotein 38, and suggesting its role in remodeling damaged tissue.^63^ Furthermore, this study indicated that FGF-2 was the major ERK-activating factor released after injury. Additionally, FGF is known to affect proliferation, by either increasing or decreasing it, depending on the receptor involved.^64,65^ Indeed, the effects of FGF-2 on cartilage are twofold: it can promote cartilage repair when binding to FGFR3, but it can also contribute to cartilage degradation when binding to FGFR1. Yan et *al.* proposed that FGF-2 triggered the degradation of proteoglycans in cartilage and found that FGF-2 could inhibit the long-term accumulation of proteoglycans in articular chondrocytes in both *in vitro* and *in vivo* studies.^66^ Moreover, Ji and colleagues demonstrated that FGF-2 promotes OA by suppressing miR-105, leading to the upregulation of Runx2 and ADAMTS, critical enzymes involved in ECM degradation.^67^ Further studies are required to clarify the role of FGF-2 in the equine cartilage response to overloading, particularly its involvement in the upregulation of proteases and the inhibition of proliferation observed following MS. Nevertheless, in our study, the lower ECM content in cartilage-like *in vitro* models following MS correlated with increased FGF-2 release and upregulation of *RUNX2* and *ADAMTS5*, suggesting their involvement in ECM degradation.

In conclusion, our study highlights the complex relationship between MS and cartilage metabolism, showing that mechanical stress can induce osteoarthritic features such as inflammatory mediators, ECM downregulation, and proteinase upregulation in a cartilage-like *in vitro* model derived from BM-MSCs. Furthermore, these results underscored the importance of controlled mechanical loading in cartilage models, as excessive compression can mimic the degenerative changes seen in joint diseases like OA. Many mechanosensors are located at the cell surface, including the primary cilium, various ion channels such as TRPV4 and PIEZO, and integrins, as reviewed.^21,22,68^ These mechanosensors transduce mechanical signals into intracellular cues, which depend on the magnitude of the MS and can be modulated by the surrounding microenvironment. Additionally, MS can directly or indirectly contribute to the upregulation of proteases that modify the ECM leading to the release of various factors that influence cell behavior. Consequently, further studies are needed to fully elucidate the mechanisms linking the MS to osteoarthritic features in our cartilage-like models. Although further research is needed to better understand the mechanotransduction mechanisms, this study lays the groundwork for utilizing MS and MSC-derived cartilage-like *in vitro* model to simulate certain aspects of OA. Eventually, the *in vitro* model we propose herein offers new perspectives for developing therapeutic strategies aimed at protecting cartilage from degradation, slowing OA progression, and advancing cartilage repair approaches.

## Supplementary Material



## Notes

(Figure S1) Evaluation of cell viability upon 7 days of mechanical stimulations and (Figure S2) effect of mechanical stimulations on type X collagen (PDF)
