# Supplementary material for: Mechanical Stimulation of Equine Bone Marrow Mesenchymal Stromal Cell-Derived Cartilage-Like In Vitro Model Triggers Osteoarthritis Features
Source: ACS Biomater Sci Eng. 2025 Jun 13;11(7):4153–65. doi: 10.1021/acsbiomaterials.5c00500 (PMC12264857; doi:10.1021/acsbiomaterials.5c00500)
Supplement: Supplementary file 1 [file ab5c00500_si_001.pdf]

Mechanical stimulation of equine bone marrow mesenchymal stromal cells-derived cartilage-like in vitro model triggers osteoarthritis features

*Romain Contentin, Cassie Jehl, Kevin Commenchail, Florence Legendre, Philippe Galéra, Frédéric Cassé † and Magali Demoor †**

† These authors contributed equally.

AUTHOR INFORMATION

Université Caen Normandie, Normandie Univ, BIOTARGEN UR7450, Normandie Equine Vallée, GIS CENTAURE F-14000 CAEN, FRANCE

CORRESPONDING AUTHOR

magali.demoor@unicaen.fr

The supporting information file consists of two pages, including the cover sheet and the supplementary figures (Figure S1&S2).

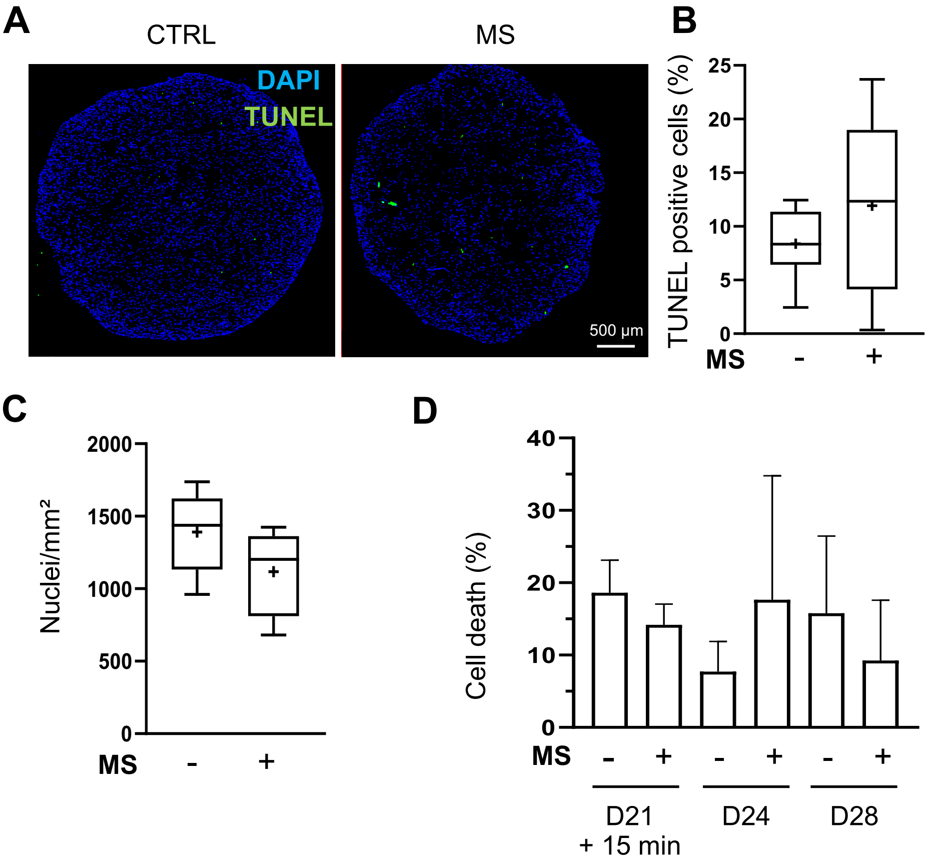

Figure S1. Mechanical stimulations decrease MSC-derived chondrocytes proliferation without impact on cell death

BM-MSCs were seeded at passage 4 in collagen sponges (5 x 10^5 cells/sponge) and cultured for 21 days in a chondrogenic medium to produce a cartilage-like organoid. (A) Micrographs of EdU positive cells from organoids cultured in static condition (-) or subjected to mechanical stimulations (MS) for 7 days (15 min compression under 2.5 kPa at 0.2 Hz every 36 h, n=6). (B) Box plot of the number of TUNEL positive cells and DAPI-stained nuclei (C) (mean (cross), median (line), ± SD error bars, n=6). (D) Histogram of cell death evaluated in media by a cytotoxicity assay (toxilight assay) (n=4). The Mann Whitney test was used to analyze differences compared to the corresponding static condition.

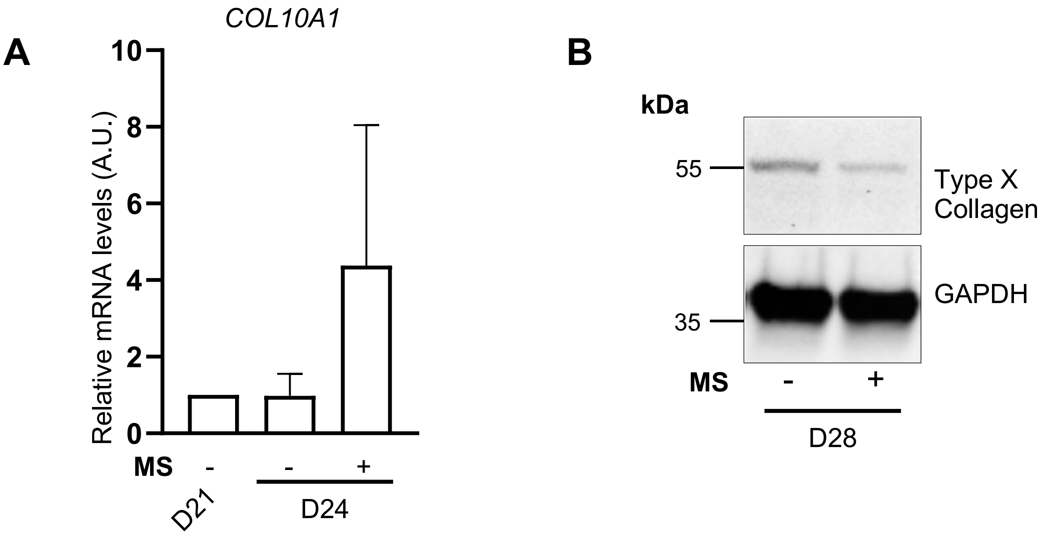

Figure S2. Effect of mechanical stimulations on type X collagen
BM-MSCs were seeded at passage 4 in collagen sponges (5 x 10^5 cells/sponge) and cultured for 21 days in a chondrogenic medium to produce a cartilage-like in vitro models. The cartilage-like in vitro models were then cultured in static condition (-) or subjected to mechanical stimulations (MS +) for 3 days (D24) or 7 days (D28) (A) Histogram of the relative mRNA levels of type X collagen (n=3). Total mRNA was extracted and RT-qPCRs were performed to assess the transcript level of type X collagen as shown in the panel. β-ACTIN and PPIA were used as reference genes. The Mann Whitney test was used to analyze differences compared to the corresponding static condition. (B) Representative immunoblots of the type X collagen and GAPDH proteins (n=2).
